# The energy divide: Integrating energy transitions, regional inequalities and poverty trends in the European Union

**DOI:** 10.1177/0969776415596449

**Published:** 2016-07-26

**Authors:** Stefan Bouzarovski, Sergio Tirado Herrero

**Affiliations:** The University of Manchester, UK

**Keywords:** Energy poverty, energy transition, European Union, prices, regional inequalities

## Abstract

Energy poverty can be understood as the inability of a household to secure a socially and materially necessitated level of energy services in the home. While the condition is widespread across Europe, its spatial and social distribution is highly uneven. In this paper, the existence of a geographical energy poverty divide in the European Union (EU) provides a starting point for conceptualizing and exploring the relationship between energy transitions – commonly described as wide-ranging processes of socio-technical change – and existing patterns of regional economic inequality. We have undertaken a comprehensive analysis of spatial and temporal trends in the national-scale patterns of energy poverty, as well as gas and electricity prices. The results of our work indicate that the classic economic development distinction between the core and periphery also holds true in the case of energy poverty, as the incidence of this phenomenon is significantly higher in Southern and Eastern European EU Member States. The paper thus aims to provide the building blocks for a novel theoretical integration of questions of path-dependency, uneven development and material deprivation in existing interpretations of energy transitions.

## Introduction

The inability of many European households to access or afford an adequate level of energy services in the home is gaining increasing academic and policy attention across the continent (Bouzarovski, 2014). This condition, described as either energy or fuel poverty (Boardman, 2009; Bouzarovski et al., 2012; Li et al., 2014), has been recognized by the European Union’s (EU’s) ‘Third Energy Package’ in relation to the concept of ‘vulnerable consumers’ (see Directives 2009/72/EC and 2009/73/EC on the liberalization of gas and electricity markets and Vulnerable Consumer Working Group (VCWG), 2013). Also of note are two opinions by the European Economic and Social Committee (EESC), drawing attention to the energy poverty implications of the economic crisis and of the liberalization of national energy markets (EESC, 2011) while calling for co-ordinated action to prevent and address energy poverty in the EU (EESC, 2013). Such initiatives are all the more prescient in light of the far-reaching economic, social and infrastructural reconfigurations brought about by on-going low-carbon and institutional energy transitions across Europe (Bridge et al., 2013).

Efforts to study the dynamics of energy poverty at the scale of the EU have been making an important contribution to such debates (Bouzarovski, 2014; Braubach and Ferrand, 2013; Healy, 2004; Healy and Clinch, 2004; Thomson and Snell, 2013). Work in this vein has identified a number of household-level factors that influence the likelihood of experiencing domestic energy deprivation, including income, socio-demographic characteristics, dwelling typology and age, tenure status and rural versus urban location. Substantial differences among EU Member States have been detected, with Southern and Eastern European countries generally reporting a higher incidence of energy poverty. At the same time, policy organizations and advocacy groups have emphasized the existence of a social ‘energy divide’ across the EU, with deprived households in most Member States being unable to meet their basic energy needs, while being penalized by high and increasing energy costs due to the combination of rising prices and inefficient properties (National Energy Action, 2014). Pan-European energy poverty research has been largely enabled by the Eurostat agency’s compilation of a rich body of statistics on poverty and social exclusion, including data on the inability to keep one’s home adequately warm, arrears in utility bills and other objective housing indicators of domestic energy deprivation. This initiative started in 1994 with the European Community Household Panel (ECHP), and has been developed further since 2003 via the Survey on Income and Living Conditions (EU-SILC).

Although EU-SILC-sourced information on monetary poverty and material deprivation has provided a pivotal point for the formulation of energy poverty indicators, a more detailed investigation of the spatial patterns embedded within EU-SILC energy poverty data at the scale of Member States is still lacking. Moreover, it is unclear whether the substantial differences among mean domestic energy prices in the EU (Fiorio and Florio, 2013) are also associated with varying rates of domestic energy deprivation. Gaps of knowledge also exist with regard to the understandings of changes in monetary deprivation, material hardship and energy poverty trends as a result of the Euro crisis, which has allowed agglomeration economies (seen as the benefits that firms accrue from geographical concentration, see Creutzig et al., 2014) to benefit a limited set of countries at the expense of others, despite the existence of a unified economic area. In broader terms, there is a need for understanding how macro-scale spatial patterns of energy poverty relate to wider economic disparities within the European realm (Petrakos et al., 2011), where the traditional notion of a core–periphery distinction (Amin, 1974; Copus, 2001) is being increasingly replaced with more nuanced polycentric accounts of inter-country differences in development paths (Featherstone and Kazamias, 2014). Further increasing these complexities have been the structural changes in the nature of economic and technological circulations brought about by energy transitions – diverse processes involving the reconfiguration of the energy sector towards new technical or institutional arrangements mainly predicated on low-carbon sources (Bridge et al., 2013; Rutherford and Coutard, 2014).

Given such lacunae, this paper explores the relationship between European energy transitions and existing socio-economic and regional inequalities, via the lens of spatial and temporal variations in the incidence of energy poverty. In contrast to conceptualizations of energy reconfigurations as one-directional processes involving a distinct set of social and technological adjustments (Den Butter and Hofkes, 2006; Geels and Schot, 2007), our principal claim is that energy transitions are predicated upon the articulation of ‘multiple transitions’ (Sýkora and Bouzarovski, 2011) involving diverse material and political sites. Moreover, we argue that European energy transitions have deepened existing regional inequalities at the macro-scale as they relate to energy poverty and similar forms of deprivation, due to the embeddedness of such processes in incumbent spatial and institutional systems. Thus, questions of path-dependency and uneven development (understood as relatively separate dimensions in previous geographical theorizations of energy transitions, such as that of Bridge et al., 2013) need to be brought together in a unified conceptual framework, in which past trajectories of change are seen as crucial determinants of the spatial outcomes of energy transition processes.

The paper has two specific objectives within these overarching aims. Firstly, we explore macro-regional differences across the EU as they relate to existing regional inequalities. The paper formulates an ‘energy poverty index’ that incorporates various material deprivation dimensions. These are subsequently cross-referenced with monetary deprivation measures. Secondly, the paper examines the relationship between the evolution of domestic energy prices, on the one hand, and income and energy poverty rates, on the other, with the aim of shedding light on the impact of the post-2008 economic crisis on households’ well-being from a domestic energy deprivation perspective, while investigating some of the complexities that underpin the expansion of inadequate residential energy serviced in Southern and Eastern European states in particular.

## Methods and data sources

The evidence presented in this paper is based on a comprehensive review of Eurostat datasets. We undertook the work in order to produce a descriptive statistical analysis of the spatial disparities and temporal patterns of indicators that have conventionally been seen as indicators of energy poverty, including domestic energy prices, welfare and deprivation in monetary and material terms. Descriptive statistics were complemented with a bivariate analysis aimed at identifying factors that exhibit a linear correlation with energy poverty incidence rates across the EU.

A few weaknesses in this data source need to be taken into consideration. Unlike other similar studies that have relied on household-level microdata for the quantification of energy poverty levels in the EU (Thomson and Snell, 2013), our descriptive and correlation analyses were conducted using individual Member States as a sampling unit, and thus the maximum yearly sample size is 28. This approach is nevertheless consistent with the scale of our analysis, which was aimed at establishing patterns across Member States as a whole. Also, the EU-SILC consensual energy poverty indicators rely on households’ self-reported assessments of their domestic energy affordability strain, which has received some criticism in the literature (Healy, 2004; Petrova et al., 2013; Thomson and Snell, 2013). Yet, EU-wide information about energy poverty rates based on household income and expenditure is not available, and this allegedly objective alternative is not exempt of methodological problems either (Healy and Clinch, 2004; Heindl, 2013). At the same time, our analysis was limited to electricity and gas prices because Eurostat statistical information is not widely available with respect to less conventional energy carriers, such as district heating, firewood or coal. Nevertheless, gas and electricity were jointly responsible for more than two thirds of household energy consumption in the EU-28 as measured by the weights that constituted the Harmonized Index of Consumer Prices (HICP) in 2012 (European Commission, 2014). With the exception of Greece, Lithuania and Latvia, electricity and gas accounted for more than half of the HICP in all EU countries (European Commission, 2014).

## Landscapes of socio-technical transition and energy inequality in the European Union

In contrast to the almost complete lack of policy and research during the 1990s, energy poverty has recently become the subject of intense practical and scientific scrutiny. The public recognition of the problem commenced in the UK and Ireland, where it was largely seen within the context of debates on fuel poverty. Emergent discussions of the drivers and consequences of energy poverty have focused on European countries, such as France (Derdevet, 2013; Devalière, 2013; Dubois, 2012), Germany (Billen, 2008; Heindl, 2013; Kopatz, 2009; Tews, 2014), Spain (Bilbao and Castro, 2013; Tirado Herrero, 2012; Tirado Herrero et al., 2014), Austria (Brunner et al., 2012), Italy (Miniaci et al., 2008; Valbonesi et al., 2014) and Greece (Dagoumas and Kitsios, 2014; Katsoulakos, 2011; Santamouris et al., 2007, 2013), as well as the newer Member States in Central and Eastern Europe (CEE) (Bouzarovski et al., 2012; Buzar, 2007a, 2007b; Fankhauser and Tepic, 2007; Kovačević, 2004; Petrova et al., 2013; Ruggeri Laderchi et al., 2013; Tirado Herrero and Ürge-Vorsatz, 2012). In post-communist countries, the number of inadequately heated homes has seen a dramatic expansion during the past two decades due to the combination of, inter alia, rapid price rises, inadequate social protection and low residential energy efficiency.

Energy transitions, therefore, are unfolding against the background of marked differences across and within EU Member States. The origins of the far-reaching reconfiguration of energy systems that is currently underway in the EU can be traced back to the 1960s. If until the last decade it was mostly driven by privatization, liberalization and Europeanization policies (Verbong and Geels, 2007), the incorporation of environmental and security concerns has expanded its original scope. Energy transitions now involve the movement ‘towards a more sustainable energy system characterized by universal access to energy services, and security and reliability of supply from efficient, low-carbon sources’ (Bridge et al., 2013). Much of the literature on the subject has focused on the temporal and sequencing aspects of the process, as evidenced by the abundance of scenario-based forecasts that assess the uptake and phasing out of energy technologies at global, regional and national scales in forthcoming decades (European Commission, 2011; International Energy Agency (IEA), 2014; Schmid et al., 2013). Nevertheless, a distinct strand of work has started to address the geographical components of energy transitions. Authors working in this vein have highlighted, inter alia, concepts of place, scale and territory in acknowledging the ‘spatially constituted’ character of energy system reconfigurations (Bridge et al., 2013).

The EU offers an unprecedented opportunity for interrogating the spatial inequalities that underpin energy transitions, not the least due to the bloc’s diverse economic and social geography, as well as its leadership role and declarative commitment towards climate change mitigation targets. However, it is increasingly becoming clear that a single energy transition does not exist across Europe, as the nature of restructuring trends in this sector is contingent upon local and national circumstances. Thus, Northern and Western countries have mostly focused on the decarbonization of the economy, although different paths have been followed within this general undertaking. Germany’s ‘Energiewende’ can be seen as a prime example of the large-scale uptake of renewable and energy-efficiency technologies (Gullberg et al., 2014; Scholz et al., 2014), while states such as the UK have relied on more conventional and centralized low-carbon technologies. In the South, pioneering governments in the uptake of renewable technologies have recently become subject to interrupted development trajectories due to the scaling back of support mechanisms within the context of austerity and fiscal consolidation packages. Such is the case of Spain and Italy, where investment in clean energy has dwindled in recent years (European Trade Union Institute, 2013), even though renewables have been proposed as way of overcoming both climate change and Euro crisis challenges in this region (Creutzig et al., 2014). In the former socialist states of CEE, the energy transition has primarily taken the form of policies aimed at the liberalization and privatization of the energy sector. These have unfolded within the context of a wider set of economic and institutional transformations that has been underway since the 1990s (Bouzarovski, 2009).

Issues of domestic energy deprivation are often missing or insufficiently incorporated in this landscape of multiple transitions brought about by energy reconfiguration processes. Numerous interactions, synergies and trade-offs between environmental and social objectives have been described at the nexus between climate change mitigation and energy poverty (Ürge-Vorsatz and Tirado Herrero, 2012). Such is the case of market-based mechanisms such as carbon pricing and renewable feed-in tariffs, whose implementation can be challenging in Member States with high levels of deprivation. Equally, energy poverty-related changes in household energy consumption patterns are effectively reconfiguring the primary energy supply mix of national domestic sectors and setting new conditions for the unfolding of the energy transition. This includes the movement away from ‘modern’ energy carriers (electricity and gas by firewood and coal) that is currently taking place in countries such as Bulgaria (Bouzarovski et al., 2012), Hungary (Tirado Herrero, 2013) and Greece (Knight, 2014; Lekakis and Kousis, 2013).

At the same time, scholarship on energy transition processes as they relate to urban, regional and national inequalities has opened important conceptual avenues for understanding the socio-spatial tensions that accompany processes of technological change. There is a growing recognition that spatial formations both influence and are shaped by energy transitions – this is particularly visible at the local level, where place-based systems of provision can challenge wider institutional norms and infrastructural arrangements (Rohracher and Späth, 2014). It has also come to be accepted that energy transitions are not homogeneous, singular and consensual pathways (Rutherford and Coutard, 2014: 1368) towards clear end-states, but rather involve manifold shifts across time and space (Fouquet and Pearson, 2012) that are multi-dimensional and enacted (Turnheim and Geels, 2012). Nevertheless, how these different dimensions influence patterns of social deprivation in the context of wider spatial differences and path-dependencies remains unclear. In the EU context, there is a need for unpacking the manner in which energy transitions are being shaped by national-level policy specificities while interacting with existing patterns of regional and social inequality.

## Energy poverty ‘regions’ in the European Union

Previous research has established significant differences in the incidence and characteristics of energy poverty across the EU. Higher levels of self-reported indoor thermal discomfort were found for Southern Member States in the 1990s (Healy, 2004) and in the 2000s (Thomson and Snell, 2013), as a result of the poor efficiency and lack of adequate heating systems in the housing stock of these countries. Later work has confirmed the paradox involving EU members in the Mediterranean basin: even though winters are milder in countries such as Portugal, Spain, Italy, Malta, Greece and Cyprus, these countries recurrently report high percentages of people who are unable to keep their home warm. Such states have consistently found themselves above the EU average when it comes to the value of key domestic energy deprivation indicators. The Euro crisis, with its rapid increase in unemployment and income inequality, has further exacerbated this situation.

Nevertheless, CEE states have recorded Europe’s highest energy poverty levels. The vulnerability of citizens in countries such as Estonia, Lithuania, Latvia, Poland, Czechia, Slovakia, Hungary, Slovenia, Croatia, Romania and Bulgaria can be attributed to the legacies of the centrally planned economy, such as the poor thermal insulation properties of the housing stock, the presence of historically low energy prices and the predominance of an unsustainable supply mix. The transition to a market economy in the 1990s added to these issues by bringing about the upward rebalancing of energy tariffs without the development of adequate social welfare and energy-efficiency mechanisms. Institutional inertia exacerbated antecedent difficulties, alongside the dependence on Russian energy imports and associated infrastructural lock-ins (Bouzarovski, 2010, 2014; Hiteva, 2013; Kovačević, 2004; Ürge-Vorsatz et al., 2006).

Energy poverty is also present in Western and Northern European Member States: Ireland, UK, France, Belgium, Germany, Austria; as well as the Netherlands, Luxembourg, Denmark, Sweden and Finland to a much lesser extent. In such countries, the issue tends to be restricted to specific demographic groups or types of housing. It is thus principally linked to the inability to purchase ‘affordable warmth’ (Boardman, 2010) among low-income households living in energy-inefficient homes. While energy poverty rates have been shown to be significant in the UK, Ireland, France and Belgium, the problem is less pervasive in other countries within this geographic grouping.

Existing knowledge thus suggests a macro-regionalization of the EU in three clusters of countries with different energy poverty levels and dynamics. In order to explore the consistency of this categorization with the respect to correlation analysis presented in the previous section, we plotted the average value of Eurostat’s monetary deprivation indicator ‘at-risk-of-poverty’ rate (percentage of the population with an income below 60 per cent of the national median, after social transfers) against an ad-hoc composite energy poverty index for each member state. The energy poverty index took into account the EU-SILC population percentages of people who have reported (i) being unable to keep their homes adequately warm (*Inability*); (ii) having arrears in utility bills (*Arrears*); and (iii) living in a home with a leaking roof, or the presence of damp and rot (*Housing faults*):

$$\begin{array}{l} \mathrm{Energy poverty index}=\;(0.5\;\times \;\%\;Inability\;+\;0.25\;\times \\ \%\;Arrears\;+\;0.25\;\times \;\%Housing\;faults)\;\times \;100 \end{array}$$

In the index, the indicator *Inability* receives a higher weight in order to reflect the greater importance that our assessment gives to self-reported thermal discomfort levels in comparison with the indicator *Arrears*, which keeps track of late payment levels in energy and other utility bills. At the same time, *Housing faults* is closely related to, but not necessarily a direct indicator of, energy poverty. Our weighted values approach is based on previously developed energy poverty indices and weight values^1^ (Healy, 2004; Thomson and Snell, 2013). It is based upon the premise that consensual measures (such as the self-reported inability to keep warm) are insufficient to capture the complex economic and material underpinnings of energy poverty, and should be combined with indicators describing the housing and financial conditions of the population in order to obtain a fuller picture (Bouzarovski, 2014; Dubois, 2012).

**Figure 1.**
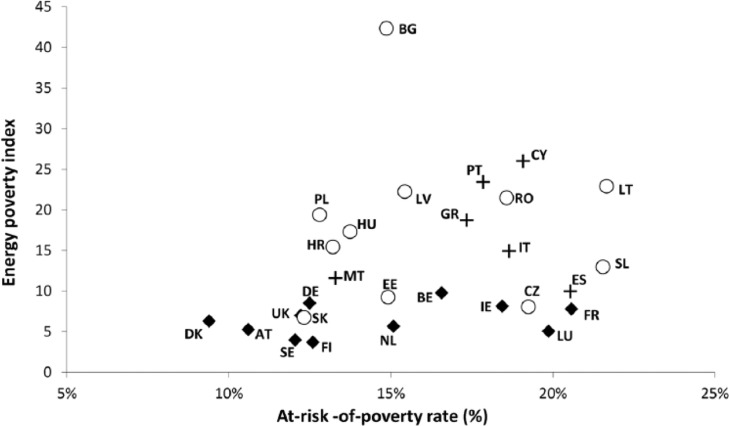
Percentage of people at risk of poverty versus the energy poverty index. Average for European Union member states for 2003–2013 for both variables.

The results of the bivariate comparison (Table 1) show a low degree of positive linear correlation between the energy poverty index and the at-risk-of-poverty rate, even though relatively high levels of positive and statistically significant linear correlations exist on an indicator-by-indicator basis. In terms of three regions identified for the spatial analysis of energy poverty trends in the EU (Figure 1), Western and Northern countries (noted in black diamonds) belong to a compact cluster reporting low energy poverty levels in relation to the at-risk-of-poverty rate. At the same time, Southern (crosses) and CEE Member States (circles) form a more heterogeneous group. They are characterized by energy poverty index values that are higher in relation to their at-risk-of-poverty-rates. With respect to the measurement of poverty and social exclusion, these results highlight the importance of material and housing deprivation dimensions, such as the inability to keep the home adequately warm. They point to the need for moving beyond purely monetary indicators, such as the at-risk-of-poverty rate.

**Table 1.** Correlation matrix: Pearson’s *r* coefficients of linear correlation between Survey on Income and Living Conditions (SILC) energy poverty indicators and index (columns) and the at-risk-of-poverty rate (rows), calculated upon average values of EU-28 Member States for the period 2003–2013.

	Inability	Arrears	Housing faults	Energy poverty index
At-risk-of-poverty rate (after social transfers)	.523**	.574**	.480**	.264

** *p* < 0.01; * *p* < 0.05 level.

The analysis thus suggests that a core versus periphery distribution is a better descriptor of the spatial disparities in energy poverty rates across the EU than the traditional three-region model. Western and Northern Member States have generally fared far better than Southern and CEE Member States in terms of domestic energy deprivation. This can principally be attributed to the higher macroeconomic performance and income levels among the latter, as well as the improved condition of the housing stock and more effective targeting of vulnerable groups. Overall, the principal differences between core and periphery countries are reflected in the degree of public recognition received by energy poverty, its socio-demographic extent and the structural drivers of the condition (see Table 2). At this point, it should be emphasized that the core–periphery distinction should not be seen in binary terms: substantial differences can be found among individual Member States at the periphery, suggesting that national, regional and local conditions matter more in this more disadvantaged cluster of EU countries. However, the relatively high degree of systemic similarities in the underpinnings and driving forces of energy poverty in the periphery also justifies the treatment of CEE and Southern European states as part of a unified geographical category in this context.

**Table 2.** A typology of energy poverty factors and implications as they vary along the core–periphery axis in the European Union.

Macro region	Core countries in Western and Northern Europe	Periphery in CEE and the Mediterranean
Public recognition	Well-established in the UK and Ireland, officially and widely acknowledged in France. Less visible in other countries.	Historically limited public recognition, recently rising to the top of the social agenda in austerity-hit countries.
Principal drivers	Low incomes, high energy prices, inefficient homes, disproportionately high energy needs.	Variable by country. Largely same as core countries but also involving questions of housing tenure and infrastructural access to adequate energy sources.
Socio-demographic extent	Typically concentrated within a limited section of the population with energy affordability problems.	A systemic condition, affecting both low- and middle-income strata.
Relationship with energy transitions	Energy poor households have been adversely affected by price increases associated with low-carbon energy transitions, but are benefiting from energy-efficiency improvements associated with the process.	Dynamics of crisis-induced austerity and post-communist transformation are adding new levels of complexity to the energy poverty implications of low-carbon transitions, which are themselves less pronounced in this region.

CEE: Central and Eastern Europe.

## Domestic energy prices: drivers and descriptors of energy poverty

### The role of EU policies

Increases in domestic energy prices have long been regarded as the crucial underpinning of energy poverty. The EU is a world region highly dependent on imports of primary energy sources. The bloc’s sensitivity to wider trends in global and regional commodity markets, means tghat increasing energy prices are an issue of significant concern among relevant institutions. The far-reaching impact of energy tariffs on household well-being and the competitiveness of EU economies is now widely recognized (European Commission, 2014). From the perspective of final residential energy users, evidence indicates that the price of domestic energy in the EU has consistently increased at faster-than-inflation rates at least since the mid-1990s, progressively reducing the purchasing power of households unless compensated by deflation in other domestic consumption categories.

The observed evolution of energy prices needs to be seen not only as a consequence of international commodity market trends and national conditions, but also within the context of multiple energy transitions. The first of such processes is the transformation of the energy sector, a process that started in the 1990s and has consisted of the privatization of publicly owned utility companies, the ‘horizontal’ and ‘vertical’ unbundling or vertical disintegration of network activities and the liberalization and opening of markets for competition (Florio, 2013). Even though these measures were meant to deliver increased levels of competition and a reduction in end-use prices, it is now clear that regulatory reforms have not always achieved the desired results, especially when it comes to domestic energy tariffs, consumer welfare and satisfaction levels, as well as households’ ability to pay bills on time (Fiorio and Florio, 2008; Poggi and Florio, 2010; Pollitt, 2012). In the post-socialist states of CEE, ambitious policy packages based on the privatization of utilities were introduced in the 1990s by international financial institutions. Such measures were put in motion with the declarative aim of preventing the collapse of the energy supply infrastructure following the downfall of central economic planning, and addressing the structural inefficiencies inherited from the previous system. Failures in the successful execution of this process have been attributed to the emergence of substantial legal and policy obstacles, as well as fierce resistance from consumers facing rising energy costs and rapidly declining incomes (Lampietti et al., 2007; Ruggeri Laderchi et al., 2013).

A second relevant trend is the decarbonization of energy systems – a large-scale policy effort driven, inter alia, by EU institutions. The process has been motivated not only by environmental concerns and climate commitments, but also by the substantial energy import dependency levels of many Member States. However, low-carbon policies have not been neutral in energy poverty terms, mainly because they have entailed the development of mechanisms for internalizing the social costs of carbon emissions. With carbon prices generated via the EU Emissions Trading Scheme (EU ETS) being passed onto final consumers (Aatola et al., 2013; Kim et al., 2010), such policy mechanisms are affecting not only the price of domestic energy but are also influencing a range of other goods and services for which energy is a production input. Low-carbon policies in the EU are also resulting in substantial investment in the renewable energy sector, especially in solar and wind electricity (European Commission, 2014). The costs of these undertakings have also been borne by final consumers through energy bills.

There is evidence to suggest that the distributional impacts of low-carbon policies are highly contingent on issues such as household size, location and the nature of consumption, rather than income (Dresner and Ekins, 2006; Gough, 2013; Haug et al., 2010). For example, a carbon tax may have almost no regressive impacts at all, depending on the evaluation method used (as demonstrated, for example, by Martini, 2009; Tiezzi, 2005). However, placing the tax burden onto electric bills often highly disproportionately affects low-income households (Poltimäe and Võrk, 2009). This is particularly true in a number of CEE and Mediterranean countries, where urban populations rely on electricity for a range of domestic energy services; a similar situation can be found among a select group of households in the UK, where many environmental levies have been loaded onto electricity bills. The relationship between low-carbon transitions and social welfare is also reflected in improvements of household energy efficiency. In Europe, these have been particularly felt in the domain of space heating, although all types of energy demand have benefited (European Commission, 2014). Yet, even if household electricity and gas consumption in the EU declined by, respectively 1 and 15 per cent between 2008 and 2011, household energy costs have increased during the same time. The European Commission (2014) maintains that ‘low refurbishment rates of inefficient housing and replacement rates of inefficient equipment have not been sufficient to offset rising prices’ (p.10).

Germany’s ‘Energiewende’ thus provides an important focus of attention not only because it is being carried out by the largest EU member state, but also because Germany is spearheading the EU’s transition to a low-carbon, renewable energy-based economy. From an energy poverty perspective, rising surcharges for financing renewable electricity under the German Renewable Energy Sources Act (EEG) have been found to be regressive: no compensation mechanisms are provided to low-income households affected by the policy, even though exemptions have been granted to power-intensive manufacturing companies and rail operators (Grösche and Schröder, 2014; Neuhoff et al., 2013). In that sense, the German case can be seen as an early example of the potential trade-offs between environmental and social policies that have started to emerge across the EU in the transition to a low-carbon energy system. The implications of decarbonization policies on domestic energy affordability is an emergent field of inquiry for energy poverty researchers (Schlör et al., 2013).

### Spatial and temporal changes in domestic energy prices and poverty across the EU

**Figure 2.**
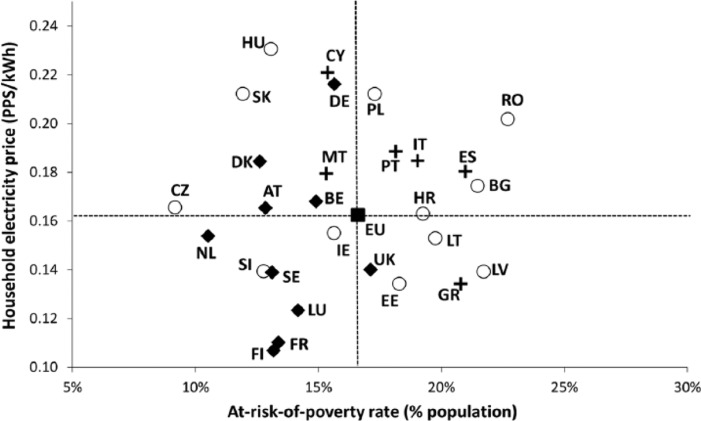
Household electricity prices (in Purchasing Power Units (PPS) as of the year 2007) versus at-risk-of-poverty rate, average for the period 2007–2013 (with a few exceptions for the poverty indicator).

**Figure 3.**
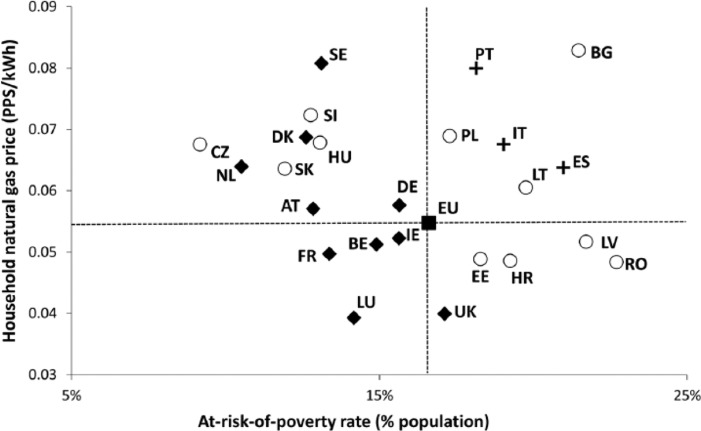
Household natural gas prices (in Purchasing Power Units (PPS) as of the year 2007) versus at-risk-of-poverty rate, average for the period 2007–2013 (with a few exceptions for the poverty indicator). Note: Cyprus, Finland, Greece and Malta missing (no data for natural gas prices).

In macro-regional terms, energy prices in the CEE space generally lie below the EU average and the values recorded for Northwestern and Mediterranean Europe (European Commission, 2014). However, Euro energy prices fail to incorporate the differences between Member States’ price levels and ‘real’ household incomes. Eurostat addresses this shortcoming by expressing prices in Purchasing Power Units (PPS): an artificial reference currency that eliminates price–income differences by correcting prices denominated in national currencies through a Purchasing Power Parity (PPP) factor, calculated on the basis of the price of a hypothetical basket of goods and services that is deemed representative of consumption patterns in individual Member States (European Communities, 2009; Eurostat, 2013). Such an approach offers a more realistic picture of the efforts that average households in different Member States need to make in order to pay for each unit of energy used at home. Prices in PPS are plotted against the percentage of people at risk of poverty (Figures 2 and 3) in order to explore the spatial variation in the exposure to these two different energy poverty factors.

The picture that arises when household prices are expressed in PPS radically alters the initial perception of cheap energy prices in worse-off countries. Thus, states with higher domestic energy prices (in PPS) are mainly located in CEE and Southern Europe, where poverty rates are also well above the EU average in most cases (see Figures 2 and 3). This imbalance is particularly visible in the case of Poland, Bulgaria, Lithuania, Romania, Croatia, Spain, Italy and Portugal: countries with above-average domestic energy prices and at-risk-of-poverty-rates for the period between 2007 and 2013.

**Figure 4.**
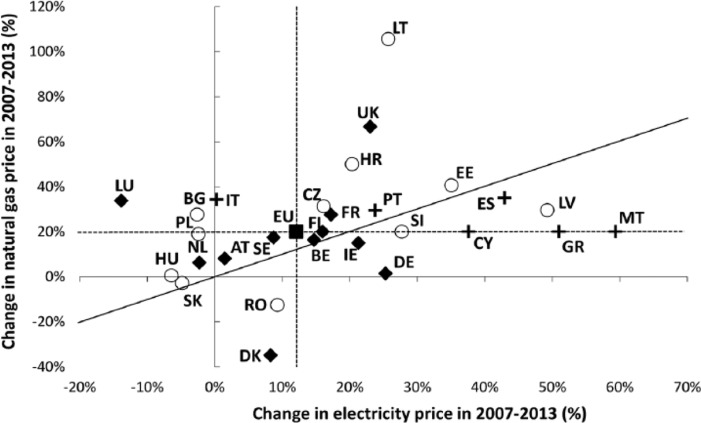
Change in household natural gas prices versus change in household electricity prices (accumulated percentage, calculated on real prices denominated in national currency) of member states, that occurred between the years 2007 and 2013. Note: (1) no household natural gas prices data is available for Greece, Finland, Malta and Cyprus; for comparative purposes, they are displayed distinctly along the line representing the average natural gas price of the European Union; (2) the diagonal line indicates a theoretical line along which natural gas and electricity prices increase at the same rate.

Having identified a general upward trend in domestic energy prices in the EU, we also assessed the evolution of household energy prices across the EU by directly estimating the rates of increase (in percentage points) in natural gas and electricity prices that occurred between the second semester of 2007 and the second semester of 2013. These figures were calculated on the basis of real prices denominated in national currencies, in order to avoid fluctuations associated with exchange rates. In the case of Member States that adopted the Euro between 2007 and 2013 (Malta, Slovakia, Cyprus and Estonia), a currency conversion was necessary prior to calculating rates of increase. Such results complement (and overlap with) the results presented in the previous section by showing trends in the key domestic energy carriers among EU households – electricity and gas. The percentage increases (see Figure 4) indicate that natural gas prices in the EU rose faster (20 per cent on average) than electricity prices (12 per cent) during the assessed period. This result is relevant from an energy poverty perspective, given the central role of natural gas in fuelling domestic energy services relevant to human health and well-being in many European countries (Fouquet, 2011). It also highlights the distinction between an energy poverty core and periphery in the EU: the citizens of Southern and post-socialist CEE member were forced to put up with increases in domestic energy prices that were above the EU average (with the notable exceptions of Slovakia and Hungary, due to local energy and price policies). Particularly steep was the rise in the three Baltic republics, as well as four Mediterranean states: Malta, Cyprus, Greece and Spain. Southeastern European states (Croatia and Slovenia), as well as Portugal, also registered significant price increases.

**Figure 5.**
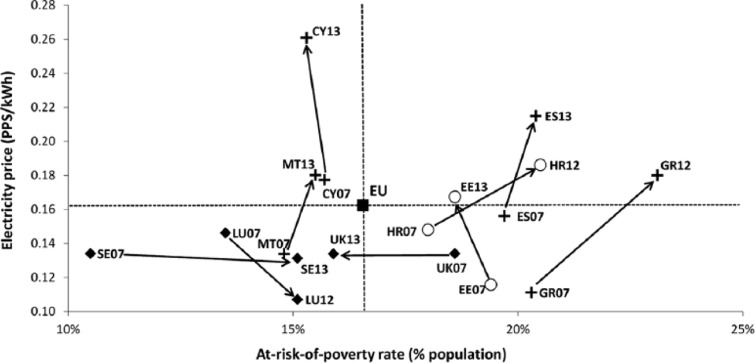
Evolution of relative positions according to electricity price (in Purchasing Power Units (PPS) as of the year 2007, per kWh) versus percentage population at risk of poverty, for selected member states between 2007 and 2013. Note: the reference European Union electricity price and poverty rate has been calculated as an average for the period 2007–2013.

**Figure 6.**
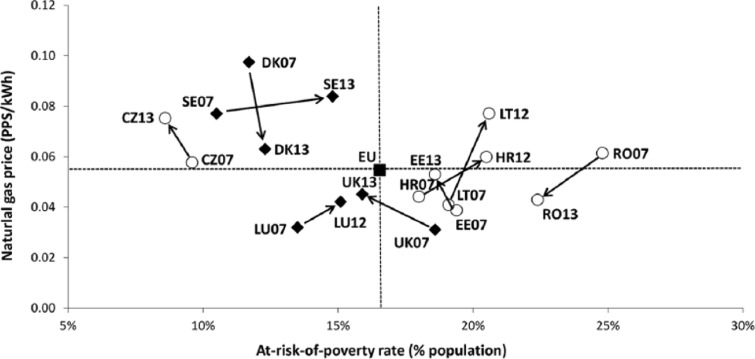
Evolution of relative positions according to natural gas price (in Purchasing Power Units (PPS) as of the year 2007, per kWh) versus percentage population at risk of poverty, for selected member states between 2007 and 2013. Note: the reference European Union electricity price and poverty rate has been calculated as an average for the period 2007–2013.

We also assessed the evolution of domestic energy prices in PPS terms. In line with the analyses above, natural gas and electricity prices in PPS were plotted separately against the at-risk-of-poverty rate. For the purpose of this exercise, we selected the eight EU countries with the largest aggregated variation (in absolute value, calculated on the percentage of change) of both energy price and poverty rates between 2007 and 2013 (Figures 5 and 6). Such comparisons allow for a synchronous visualization of the increases in energy prices and poverty levels that have occurred, in part, as a result of the Euro crisis. The outcomes of our analyses for the case of domestic electricity indicate that Member States in Southern Europe and the CEE region have been adversely affected to the greatest extent, while Northern and Western EU members have even benefitted from the transformations seen between 2007 and 2013. Within the eight Member States selected within the frame of the analysis, the citizens of Croatia, Greece and Spain are among the most vulnerable; as of 2013, over 20 per cent of the population in these countries was at risk of poverty, and had seen a substantial rise in electricity prices during the previous six years. In the case of natural gas, the former socialist states of CEE have recorded the highest changes in both the price of this domestic fuel and the monetary deprivation rate. However, unlike electricity prices, the adjustment is not one-directional; for example, Romania has reported a significant drop in the poverty rate and in the price of natural gas alike.

**Figure 7.**
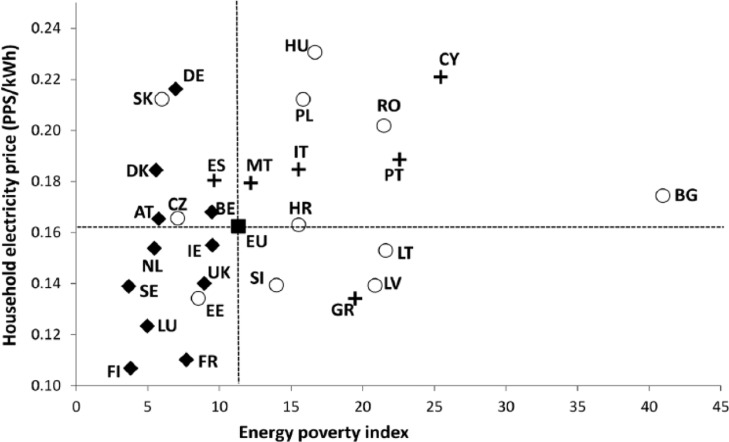
Household electricity prices (in Purchasing Power Units (PPS) as of the year 2007) versus at-risk-of-poverty rate, average for the period 2007–2013 (with a few exceptions for the poverty indicator).

**Figure 8.**
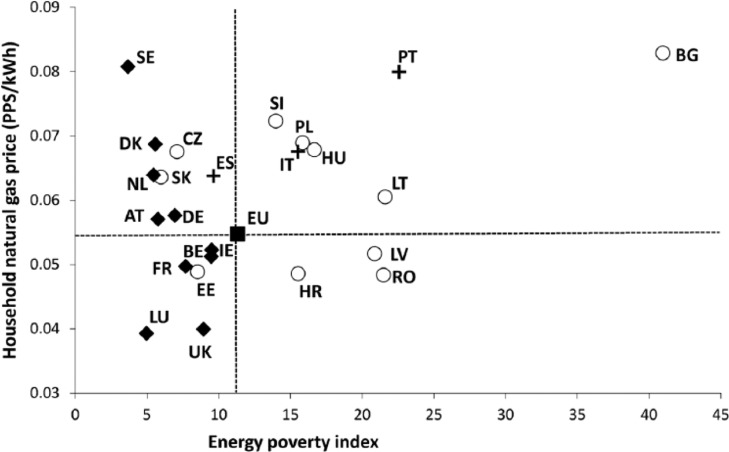
Household natural gas prices (in Purchasing Power Units (PPS) as of the year 2007) versus at-risk-of-poverty rate, average for the period 2007–2013 (with a few exceptions for the poverty indicator). Note: Cyprus, Finland, Greece and Malta missing (no data for natural gas prices).

The predominance of ‘periphery’ countries within the correlation between energy price changes and at-risk-of poverty rates indicates that the systemic forces that drive energy poverty need to be seen within the context of deeper regional disparities within the EU. While such an analysis cannot in itself demonstrate a causal link between increases of energy prices and monetary poverty levels, there is a clear clustering of countries at the nexus of these two dimensions. Even more striking results were obtained when we mapped gas and electricity prices in 2013 against the composite energy poverty index, which incorporates material deprivation dimensions (Figures 7 and 8). These analyses signal that the disproportionately high presence of domestic energy deprivation in peripheral Member States is also underpinned by wider technical and infrastructural factors. Systemically embedded economic and spatial inequalities are interacting with the diverse dynamics of energy transition to produce regionally embedded inequalities.

## Conclusions

This paper has sought to highlight the conceptual link between energy transitions and regional spatial inequalities, while emphasizing the multi-directional, spatially contingent and path-dependent nature of on-going processes of socio-technical change in the energy sector. Our theoretical approach has identified some of the key dimensions that link energy transitions and poverty via a geographical lens: the need to position energy restructuring processes within the context of existing patterns of uneven development, as well as the role that monetary and material deprivation rates play in exacerbating existing or creating new vulnerabilities. In empirical terms, the paper has involved a comprehensive assessment of the relationship between domestic energy prices and monetary deprivation rates over time and space so as to establish (i) degrees of national-scale geographic variation in energy poverty rates and (ii) the role of gas and electricity prices in shaping the temporal and spatial distribution of monetary deprivation and energy poverty.

In relation to the first aim of the paper, a cross-country and time series analysis of Eurostat data showed that there are substantial regional disparities in the exposure of various countries to the drivers of energy poverty. Our results thus challenge the findings of previous studies by suggesting that the traditional division of EU states into three clusters is increasingly replaced by a relatively well-off ‘core’ group of countries in Northern and Western Europe, and a heterogeneous ‘energy poverty periphery’ in the South and East. In the former, domestic energy deprivation is limited to specific demographic and housing groups, while the latter exhibits a more pervasive presence of the problem across a range of social strata. Thus, the notion of the ‘energy divide’ can be expanded from its original predominantly socially orientated meaning (as described in National Energy Action, 2014) to encapsulate existing inequalities in access to infrastructure services at the scale of cities, regions and countries.

Developing further our exploration of the drivers of energy poverty across Europe – and in relation to the second aim of the paper – we can conclude that domestic energy prices have consistently increased at faster-than-inflation rates for the EU as a whole since the mid-1990s. This pattern can be found throughout individual Member States, as domestic energy prices have outpaced inflation throughout the EU since 2004. Thus, state-level gas and electricity tariffs are acting on top of a more systemic piece of the energy poverty puzzle: monetary deprivation measured as the at-risk-of-poverty rate.

The peripheral region (in energy poverty terms) is highly heterogeneous, as a result of the different underlying factors involved in driving the condition – particularly when it comes to the inflationary character of domestic energy prices. In particular, the post-socialist Member States of CEE often report above-average at-risk-of-poverty rates. These have resulted in the expansion of energy poverty to a considerable degree in most countries within the region, with the notable exceptions of Czechia, Slovakia and Estonia. Paradoxically, countries in the CEE cluster have the EU’s lowest nominal energy prices (in Euro terms), but are characterized by higher-than-average energy prices when measured in PPS. Even though their real energy tariffs have not increased faster than the rest of Europe, such states are more exposed to the price factor because households spend relatively more on domestic energy than in the rest of the EU. The CEE region contains several worst case scenarios (Bulgaria, Latvia, Lithuania, Croatia and Romania) where conditions are significantly more difficult than the rest of the EU in terms of the two driving factors of energy poverty assessed in this paper: high and increasing poverty rates, and high and increasing domestic gas and electricity prices.

At the same time, Southern European Member States are also part of the energy poverty periphery due to containing higher-than-average energy poverty and monetary deprivation levels, albeit below the numbers seen in CEE. Certain trends identified in this cluster of countries stand out, however, as some countries have experienced very substantial increases in energy prices – especially for electricity – while seeing poverty levels grow after the Euro crisis and the implementation of austerity packages (especially in Cyprus, Greece, Malta and Spain). At the same time, Northern and Western Member States can be situated within the ‘core’ region identified above. They have fared better than both CEE and Southern Europe, with relatively low levels of monetary deprivation and energy poverty seen throughout. Minimal degrees of exposure to domestic energy deprivation are notable in Austria, Finland, Denmark, the Netherlands and Sweden. However, energy prices have been increasing at faster-than-inflation rates throughout the core region as well, especially in the UK.

These findings evidence the diverse geography of energy poverty in the EU, which is characterized by substantial differences among the analysed countries in terms of their exposure to the two factors analysed in the paper (monetary deprivation rates and energy prices) and their evolution. While our results do not indicate that the energy transition is leading to a radical reconfiguration of existing regional inequalities, there is evidence to suggest that the EU as a whole has experienced an increase in the levels of energy poverty as measured by EU-SILC since 2007. This highlights the need for considering – among research and policy communities alike – the differential impact that the post-2008 financial crisis is exerting on welfare levels and deprivation rates across the EU. Energy operations in countries affected by austerity and fiscal consolidation measures are of particular relevance here. There is also a necessity for acknowledging the price and energy poverty risks posed by wider energy transition processes stemming from the liberalization and privatization of the energy sector and the long-term transition to a low-carbon future.

## Notes

1. This bivariate comparison was not conducted for Eurostat’s central measure of monetary and material deprivation (‘people at risk of poverty or social exclusion’), given that that this complex metric is based, among other elements, on the indicators Inability and Arrears, and thus issues of collinearity between variables would have arisen in the correlation analysis.
